# The Relationship Between Emotion Malleability Beliefs and School Adaptation of Middle School Boarders: A Chain Mediating Effect of Psychological Resilience and Peer Relationships

**DOI:** 10.3390/bs15111444

**Published:** 2025-10-23

**Authors:** Yixuan Han, Shiyu Zheng, Xuehong Chen, Jing Zhang, Yao Meng

**Affiliations:** 1 Department of Psychology, Renmin University of China, Beijing 100872, China20226901@ruc.edu.cn (S.Z.);; 2 School of Nursing, Nanjing Medical University, Nanjing 211166, China

**Keywords:** emotion malleability beliefs, resilience, peer relationships, school adaptation

## Abstract

Middle school boarders are more prone to maladjustment to school due to a lack of parental accompaniment and long school hours. Focusing on this specific group, this study explored the effects of emotion malleability beliefs on their adjustment to school and their influential pathways, and constructed a hypothetical model with resilience and peer relationships as chain mediators. The Implicit Theories of Emotion Scale, the Adaptation to School Scale for Middle School Students, the Adolescents Resilience Scale, and the Peer Relationship Assessment Scale were applied to measure 511 middle school boarders. The results showed that there were significant positive correlations between emotion malleability beliefs, resilience, peer relationships, and adaptation to school. Emotion malleability beliefs directly influence adaptation to school and are indirectly associated with adaptation to school through the chain mediation of resilience and peer relationships. Our study emphasized the important influence of emotion malleability beliefs on boarding students’ adaptation to school, which also hinted to us that interventions targeting emotion malleability beliefs may help enhance resilience and peer relationships, thereby supporting school adaptation.

## 1. Introduction

In regions with limited educational resources, boarding schools are the primary tool for the education sector to universalize basic education, and are also an excellent means for students to obtain higher quality educational resources. A survey from China has shown that the total number of boarding students in Chinese rural middle schools is more than 16.8 million, with a 58.6% boarding rate (Wu & Qin, 2017). Good school adaptation is essential for boarding students to establish consistent study habits and to advance their physical and mental well-being (Tan & Chen, 2007). However, boarding adolescents are vulnerable to adaptation problems due to their single life and separation from their parents (Shen et al., 2021). Due to their immature values and emotions, students in junior high school, particularly, could be more negatively affected by school adaptation (Bailen et al., 2018; Eoh et al., 2022; Xie et al., 2022). Therefore, it is highly important to explore the protective factors of school adaptation in middle school boarders.

Adapting to school has been proven to be a key factor in students’ academic success and overall happiness in school. Feelings of worry, tension in interpersonal interactions, and a dread of speaking in front of others can all serve as indicators of its severity (Klizienė et al., 2018). It has been shown that students’ emotion regulation abilities affect their school adaptation (S. Huang et al., 2015). However, middle school students have difficulties in emotion control and behavioral inhibition, which would make them less likely to regulate their emotions effectively (Malagoli et al., 2022). Recent studies have shown that emotion malleability beliefs are a protective factor in emotion regulation (Ford & Gross, 2018). It refers to how much a person believes that emotions are malleable or controllable (Tamir et al., 2007), and having these beliefs is linked to experiencing more positive emotions and fewer negative emotions daily among adolescents (J. Zhang et al., 2023). Studies demonstrated that emotion malleability beliefs increased the use of cognitive reappraisal and reduced depressive symptoms (Ford et al., 2018; Goodman et al., 2021; Kneeland et al., 2016b). Thus, greater belief in emotion malleability might contribute to middle school boarders’ emotional regulation, and may influence school adaptation.

The implicit theories of emotion suggest that individuals with more emotion malleability beliefs have an incremental view of human characteristics, and provide rational explanations for subsequent emotional behavioral responses based on personality traits and environmental information (Burnette & Finkel, 2012). Based on this theory, our literature review revealed that both psychological resilience and peer relationships may play vital roles in the link between emotion malleability beliefs and school adaptation. Psychological resilience is the ability to self-regulate, adapt positively to the environment, and quickly recover from difficulties or adversities (Yu & Zhang, 2005). Individuals with greater emotion malleability beliefs are more accepting of uncertainty and use positive strategies to overcome challenges, resulting in greater psychological resilience (Congard et al., 2022; Zhu et al., 2020). Moreover, psychological resilience, as a positive personality trait, could mobilize individuals’ protective resources and help them effectively cope with adverse situations (Klika & Herrenkohl, 2013). Students with stronger psychological resilience are better equipped to overcome obstacles and demonstrate adaptability while handling pressures (Su et al., 2021). This results in a favorable prognosis of their adaptation to school life (G. Zhang et al., 2014). Therefore, it could be hypothesized that psychological resilience might mediate the relationship between emotion malleability beliefs and school adaptation.

Meanwhile, peer relationships are crucial for middle school boarders, which might mediate the link between emotion malleability beliefs and school adaptation. Students who hold greater emotion malleability beliefs perceived more interpersonal support and had higher quality social interactions (Tamir et al., 2007), possibly because these students might exhibit strong social and emotional skills, and build strong peer relationships (Z. Huang et al., 2021). Moreover, high-quality peer relationships also provide adolescents with a sense of belonging and emotional support, and help them better adapt to the school environment (Wang et al., 2018). In addition, researchers have indicated that peer rejection predicts individual learning difficulties, grade repetition, and higher dropout rates (Chen et al., 2019). Therefore, peer relationships might also mediate the link between emotion malleability beliefs and school adaptation.

Further studies have shown that individuals with greater psychological resilience have better interpersonal relationships (Halilova et al., 2020; Tamir et al., 2007). Students with greater psychological resilience are more confident in their interpersonal communication abilities (Glaser, 1998), peer relationships (Graber et al., 2016), and self-awareness (Mertens et al., 2022) than those with low psychological resilience. Thus, it could be speculated that psychological resilience may affect the school adaptation of middle school boarders by establishing positive peer relationships.

In this study, we tested how emotion malleability beliefs influence school adaptation and explored the chain mediating role of psychological resilience and peer relationships in middle school boarders. The hypotheses were as follows: (A) Middle school boarders’ emotion malleability beliefs positively predict their school adaptation. (B) Psychological resilience plays a mediating role between emotion malleability beliefs and school adaptation. (C) Peer relationships play a mediating role between emotion malleability beliefs and adaptation to school. (D) Psychological resilience and peer relationships play a chain mediating role between emotion malleability beliefs and adaptation to school. From a novel perspective, concerning the impact of emotion malleability beliefs on school adaptation, this study offered a way for middle school boarding students to enhance their emotional adjustment and improve their mental health.

## 2. Method

### 2.1. Participants

We collected data via an online questionnaires, after recruiting 511 middle school boarding students. After excluding questionnaires with response times shorter than 5 min (indicating careless responding) or longer than 30 min (indicating disengagement), 481 valid responses were retained, yielding a validity rate of 88.2%. The sample consists of 258 boys and 296 girls, with an age range of 12–16 years old (*M* = 14.04 years, *SD* = 1.07). There were 152 (31.6%) seventh graders, 143 (29.73%) eighth graders, and 186 (38.67%) ninth graders. The study was reviewed by the Ethics Committee of the Department of Psychology, Renmin University of China. All participants provided written informed consent before the study.

### 2.2. Measures

#### 2.2.1. Implicit Theories of Emotion Scale

For the measurement of the participants’ beliefs about the malleable or fixed nature of emotion, four items modified from the Implicit Theories of Intelligence Scale by Tamir et al. (2007) were used. Two items focused on malleable or controllable beliefs (i.e., “if I want to, I can change the emotions that I have” and “I can learn to control my emotions”). Two items focused on uncontrollable or fixed beliefs (i.e., “the truth is, I have very little control over my emotions” and “no matter how hard I try, I can’t change the emotions that I have”). Each item was rated from 1 (strongly agree) to 7 (strongly disagree). The total score was used to indicate one’s beliefs about whether emotions are controllable. A higher total score indicates that the individual endorsed a stronger belief that emotions are controllable. The Cronbach’s alpha coefficient for the four items was 0.75 in the current study.

#### 2.2.2. The Scale of School Adjustment for Junior High School Students

School adaptation was measured by the Scale of School Adjustment for Junior High School Students (Hou, 2016). The scale included 85 items from six dimensions, including academic adjustment, teacher–student relationship, peer relationship, emotional adjustment, school attitudes, and collective adjustment. Each item was rated on a 5-point Likert scale. The Cronbach’s alpha coefficient for the scale was 0.86.

#### 2.2.3. Adolescent Psychological Resilience Scale

The scale was compiled by Hu and Gan (2008), based on the psychological resilience process model. The psychological resilience was divided into individual strength and support in this scale. Individual strength included the following three dimensions: goal focus, emotion regulation, and positive cognition. Support included the following two dimensions: family support and interpersonal support. The scale consisted of 27 items, rated on a 5-point Likert scale. Higher scores indicated greater psychological resilience. The Cronbach’s alpha coefficient for this scale was 0.86 in the current study.

#### 2.2.4. Peer Relationship Assessment Scale

The peer Relationship Assessment Scale was compiled by Asher et al. (1984) and was adapted by Zou et al. (2007) according to the actual situation in China. This scale measures how individuals feel about themselves when interacting with others. It consists of 16 items, rated on a 4-point Likert scale. The scale includes reverse scoring items. The higher scores indicate better peer relationships. The Cronbach’s alpha coefficient for this scale was 0.89.

### 2.3. Data Processing

Data analysis was conducted using SPSS 26.0 (IBM Corp., Armonk, NY, USA). Harman’s single-factor method was employed to test for common method bias. Independent samples *t*-test and one-way analysis of variance (ANOVA) were used to examine the differences between each variable for demographic variables. Pearson correlation analysis was used to test the correlation between variables. Hierarchical multiple regression analysis and the PROCESS macro for SPSS (Hayes, 2013) were used to test the mediation effects. Model 6 in PROCESS was used to test the chain mediation effects (Bolin, 2014). The bias-corrected non-parametric percentile bootstrap was used to estimate the confidence intervals of the mediation effects. The normality of study variables was tested using the Kolmogorov–Smirnov test as well as skewness and kurtosis values. Although the Kolmogorov–Smirnov test was significant (*p* < 0.05), skewness (−0.74 to −0.34) and kurtosis (−0.10 to 1.12) indicated approximate normality.

## 3. Results

### 3.1. Common Method Bias Test

The Harman one-way method was adopted to test the common method bias, and the results showed that a total of 12 factors’ trait roots > 1, and the explained rate of the variance of the first factor was 33.34%, which was less than the critical value of 40%, indicating that the data of this study did not have serious common method bias.

### 3.2. Tests for Differences in Demographic Variables

An independent sample *t*-test was used to examine the influence of gender on the following four variables: emotion malleability beliefs, school adaptation, psychological resilience, and peer relationships. As shown in Table 1, emotion malleability beliefs differed significantly by gender, *t*(479) = −2.79, *p* < 0.001, showing that middle school boarding girls (*M* = 2.99, *SD* = 0.83) scored significantly higher than boys (*M* = 2.77, *SD* = 0.90). Psychological resilience differed significantly by gender, *t*(479)= −2.28, *p* = 0.02, showing that middle school boarding girls (*M* = 3.15, *SD* = 0.68) scored significantly higher than boys (*M* = 2.99, *SD* = 0.83) in terms of psychological resilience. Gender had no significant effects on school adaptation and peer relationships (*p*s > 0.05).

As shown in Table 2, one-way ANOVA was used to test the differences between the grades in the following four variables: emotion malleability beliefs, school adaptation, psychological resilience, and peer relationships. Students in different grades differed significantly in their adaptation to school, *F*(2, 478) = 3.44, *p* < 0.05. The results of multiple comparisons of LSD showed that the adaptation to school scores of seventh-grade students (*M* = 3.03, *SD* = 0.69) and eighth-grade students (*M* = 3.01, *SD* = 0.72) were significantly higher than those of ninth-grade students (*M* = 2.84, *SD* = 0.79), *p* < 0.05. Students in different grades differed significantly in psychological resilience, *F*(2, 478) = 3.86, *p* < 0.05, and the psychological resilience scores of seventh-grade students (*M* = 3.14, *SD* = 0.69) and eighth-grade students (*M* = 3.20, *SD* = 0.66) were significantly higher than those of ninth-grade students (*M* = 2.99, *SD* = 0.75), *p* < 0.05. Students in different grades were not different in emotion malleability beliefs (*F*(2,478) = 0.26, *p* > 0.05) and peer relationships (F(2, 478) = 0.12, *p* > 0.05).

### 3.3. Correlation Analysis

The correlations between emotion malleability beliefs, school adaptation, psychological resilience, and peer relationships are presented in Table 3. All variables significantly correlated with each other, which qualified for the mediation effects test.

### 3.4. Chain Mediation Effect Test

As Table 4 shows, we conducted a hierarchical multiple regression analysis using gender and grade as control variables. Using emotion malleability beliefs as the independent variable and psychological resilience as the dependent variable, the result of regression was found to be significant (*R*^2^ = 0.14, *F* = 26.57, *p* < 0.01). Emotion malleability beliefs had a significant positive effect on psychological resilience (*β* = 0.29, *SE* = 0.03). When peer relationship was included in the regression equation (*R*^2^ = 0.28, *F* = 47.06, *p* < 0.01), emotion malleability beliefs (*β* = 0.25, *SE* = 0.04) and psychological resilience (*β* = 0.38, *SE* = 0.05) positively predicted peer relationships. In addition, emotion malleability beliefs (*β* = 0.17, *SE* = 0.04), psychological resilience (*β* = 0.20, *SE* = 0.05), and peer relationships (*β* = 0.27, *SE* = 0.04) positively predicted adaptation to school.

**Figure 1:**
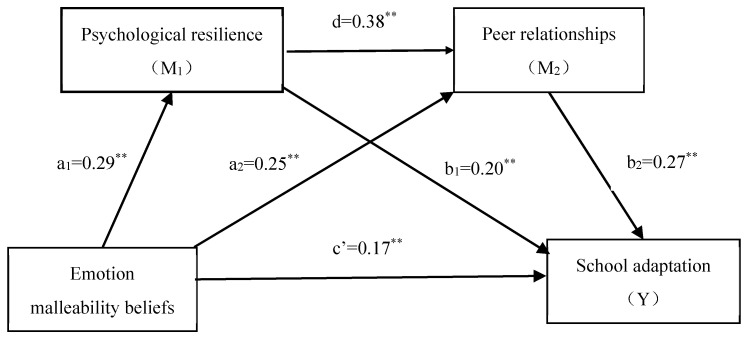
The chain mediating model of psychological resilience and peer relationships. ** *p* < 0.01.

We tested the significance of the mediating effect using the bootstrap method (see Table 5) and found the path (emotion malleability beliefs → psychological resilience → adaptation to school) to have a significant indirect effect (*β* = 0.06, 95%CI = [0.01, 0.14]). The path (emotion malleability beliefs → peer relationships → adaptation to school) also had a significant indirect effect (*β* = 0.07, 95%CI = [0.02, 0.14]), and the path (emotion malleability beliefs → psychological resilience → peer relationships →adaptation to school) had a significant indirect effect as well (*β* = 0.03, 95%CI = [0.01, 0.06]). There was a chain mediating effect of psychological resilience and peer relationships in the relationship between emotion malleability beliefs and adaptation to school. The total effect value of emotion malleability beliefs on adaptation to school was 0.33, and the total indirect effect value was 0.16, which was 48.16% of the total effect. The direct effect value was 0.17, which was 51.84% of the total effect. The chain mediating model is shown in Figure 1.

## 4. Discussion

In this study, we examined the associations between emotion malleability beliefs and school adaptation among middle school boarders. The results showed that emotion malleability beliefs were positively associated with school adaptation, with psychological resilience and peer relationships serving as mediators in this relationship. Specifically, emotion malleability beliefs were not only directly associated with school adaptation but also linked indirectly via a sequential pathway involving psychological resilience and peer relationships. These findings underscore the middle school stage as a critical developmental period for the formation of emotion malleability beliefs (Romero et al., 2014) and contribute to a deeper understanding of how these beliefs, together with psychological resilience and peer relationships, are linked to students’ adjustment in boarding contexts.

### 4.1. The Role of Emotion Malleability Beliefs in School Adaptation

We have found that the emotion malleability beliefs of middle school boarders significantly positively influence adaptation to school, which is consistent with past research showing that emotion malleability beliefs influenced fewer future depressive symptoms in individuals (De Castella et al., 2018; Ford & Gross, 2018), making them more adaptable to school life. Middle school boarders with fewer emotion malleability beliefs may feel negative emotions as uncontrollable and threatening, which may be associated with a tendency to avoid or employ negative strategies for self-protection (Kashdan et al., 2006). However, middle school boarders with more emotion malleability beliefs have greater self-efficacy in emotional regulation (De Castella et al., 2013). They are more likely to use cognitive reappraisal and actively seek solutions when facing emotional challenges, which may facilitate better adaptation to boarding school life (Schroder et al., 2018).

### 4.2. The Mediating Role of Psychological Resilience and Peer Relationships

We have found that mental resilience is a potential mediator in the relationship between middle school boarders’ emotion malleability beliefs and their adaptation to school, which is consistent with previous research. On the one hand, emotion malleability beliefs affect individuals’ mental resilience. Individuals’ intrinsic emotion-related beliefs are important factors in the enhancement and development of mental resilience (Yu & Zhang, 2005; Kneeland et al., 2016a). On the other hand, mental resilience positively influences middle school boarders’ adaptation to school. Increased mental resilience enables individuals to have positive cognitive attitudes in the face of adversity and stress, which facilitates the improvement of language skills, social skills, and academics, and mitigates the impact of stress on the individual, thus improving the individual’s adaptive capacity (Armstrong et al., 2011).

Research has shown that peer relationships may mediate the relationship between emotion malleability beliefs and adaptation to school among middle school boarders. Individuals who hold beliefs that emotions are controllable generally have greater self-efficacy that promotes the emergence and development of positive peer relationships (Ford & Gross, 2018). Furthermore, individuals holding high emotion malleability beliefs tend to have positive socio-emotional competence and are also more attentive to the feelings and thoughts of others, which also contributes to positive peer relationships (Z. Huang et al., 2021; Kendziora & Osher, 2016). According to the ecosystem theory, school is a mini society, and in this system, peer relationships are crucial, directly affecting adolescents’ growth and adjustment (X. Zhang et al., 2019). Especially in boarding schools, peer relationships become a very important source of social support for middle school boarders (Coyle et al., 2021). Positive peer relationships enable individuals to obtain more positive responses from their peers, thus helping middle school boarders to better adapt to school (Yu et al., 2017).

### 4.3. Chain Mediating Effect of Resilience and Peer Relationships

In addition, we have found that emotion malleability beliefs influence one’s adaptation to school through the chain mediating effect of psychological resilience and peer relationships. Emotion malleability beliefs are significantly positively associated with psychological resilience. In interpersonal communication, individuals with high psychological resilience quickly accept changes, use positive solutions regarding their peers, and better accept their peers, making their social support system more stable (Haddow et al., 2021). Furthermore, individuals who have positive interpersonal relationships can also receive more support from peers and can improve individual adaptability, including adaptation to school.

According to the theory of emotional reasoning, individuals tend to equate their subjective emotional experiences (e.g., “I feel anxious”) with objective facts (e.g., “therefore, the situation must be dangerous”), and this tendency can significantly exacerbate anxiety, depression, adaptation difficulties, and ruminative inner speech (Dahò & Monzani, 2025; Gangemi et al., 2021). The present study suggests that holding a belief in emotion malleability may provide boarding students with an alternative perspective, helping them to counteract the tendency for emotional reasoning. This allows them to view emotional fluctuations from a more developmental and macro-level perspective (Yeager & Dweck, 2012). When students are no longer trapped in the emotional reasoning pattern, they can mobilize greater cognitive resources to cope with stress actively. This more adaptive and flexible coping process is, in itself, a manifestation of greater psychological resilience. Students with greater resilience, as they are better able to regulate their emotions, tend to exhibit less withdrawal and aggression in social interactions (Kneeland & Dovidio, 2020). Ultimately, positive peer relationships and networks not only provide emotional support and a sense of belonging but also help students to continuously revise potential cognitive biases (including emotional reasoning) and view problems from multiple perspectives, which directly promotes their comprehensive school adaptation in academic, interpersonal, and emotional domains (Kim et al., 2024).

### 4.4. Limitations and Implications

Our study has the following limitations and suggestions for further research. Firstly, participants were drawn from a single city in Guangdong Province, and contextual factors, such as socioeconomic status and school location (urban vs. rural), were not included. These issues may limit the generalizability and nuance of the findings; future studies should involve more diverse samples and incorporate such variables as controls. Secondly, the study relied on self-report questionnaires collected at a single time point, which may introduce biases such as social desirability and common method variance. Longitudinal, multi-informant, or experimental designs are needed to clarify causal relationships among emotion malleability beliefs, school adaptation, psychological resilience, and peer relationships, and to examine whether these associations vary across developmental stages. Thirdly, although the Implicit Theories of Emotion Scale has been applied in experimental studies, it has not undergone systematic psychometric validation in Chinese junior high school populations. Future research should conduct confirmatory factor analyses and invariance testing to strengthen its validity. Finally, our study only examined the role of peer relationships in psychological resilience and students’ adaptation to school. Teacher–student and family relationships are also important components of interpersonal networks for boarding students. Thus, future studies could explore the influence of emotion malleability beliefs and psychological resilience on different interpersonal relationships, and could explore how different interpersonal relationships influence school adaptation in this chain mediating model.

Based on the identified chain mediating pathway, a tiered intervention approach may be especially effective. First, programs could focus on shaping emotion malleability beliefs, for example, through psychoeducational workshops that emphasize emotions as controllable and improvable (Kneeland et al., 2016b), thereby strengthening students’ confidence in emotion regulation. Second, resilience-training curricula could be implemented, including sessions on resilience skills, adaptive emotion regulation strategies, and social support building (Llistosella et al., 2024). Finally, by simultaneously targeting emotion beliefs, resilience, and peer relationships, schools may establish a comprehensive support system that aligns with the mediating mechanisms revealed in this study, and which contributes to healthier adjustment and well-being among boarding school students.

## 5. Conclusions

Our study provided supporting evidence for the direct influence of emotion malleability beliefs on students’ adaptation to school, and the mediating role of psychological resilience and peer relationships in the relationship between emotion malleability beliefs and adaptation to school, as well as the chain mediating effect.

## Figures and Tables

**Table 1:** Gender differences in emotion malleability beliefs, psychological resilience, peer relationships, and school adaptation (*M* ± *SD*).

Variables	Gender		*t*_(479)_	*p*
	Male (*N* = 210)	Female (*N* = 271)		
Emotion malleability beliefs	2.77 ± 0.90	2.99 ± 0.83	−2.79	0.00
Adaptation to school	2.93 ± 0.74	2.96 ± 0.75	−0.46	0.64
Psychological resilience	3.03 ± 0.74	3.15 ± 0.68	−2.28	0.02
Peer relationships	2.98 ± 0.77	3.12 ± 0.75	−1.95	0.05

Note. Differences in gender in emotion malleability beliefs, psychological resilience, peer relationships, and school adaptation were compared with *t*-tests.

**Table 2:** Grade differences in emotion malleability beliefs, psychological resilience, peer relationships, and school adaptation (*M* ± *SD*).

Variables	Grade			*F*_(2.47)_	*p*
	Seventh Grade (*N* = 152)	Eighth Grade (*N* = 143)	Ninth Grade (*N* = 186)		
Emotion malleability beliefs	2.87 ± 0.83	2.94 ± 0.88	2.87 ± 0.90	0.26	0.77
Adaptation to school	3.03 ± 0.69	3.01 ± 0.72	2.84 ± 0.79	3.44	0.03
Psychological resilience	3.14 ± 0.69	3.20 ± 0.66	2.99 ± 0.75	3.86	0.02
Peer relationships	3.04 ± 0.76	3.08 ± 0.78	3.05 ± 0.75	0.12	0.89

Note. Grade-level differences were compared with the one-way ANOVA (*F*) test.

**Table 3:** Correlation analysis of emotion malleability beliefs, school adaptation, psychological resilience, and peer relationships.

Variables	*M*	*SD*	Emotion Malleability Beliefs	School Adaptation	Psychological Resilience	Peer Relationships
Emotion malleability beliefs	2.89	0.87	1			
School adaptation	2.95	0.74	0.38 **	1		
Psychological resilience	3.10	0.71	0.36 **	0.39 **	1	
Peer relationships	3.06	0.76	0.42 **	0.44 **	0.46 **	1

Note. ** *p* < 0.01.

**Table 4:** Regression analysis of the relationships between emotion malleability beliefs, psychological resilience, peer relationships, and school adaptation.

Regression Equation		Fitting Index			Significance				
		*R*	*R* ^2^	*F*	*β*	*SE*	*t*	*LL*	*UL*
Psychological resilience		0.38	0.14	26.57 **					
	Gender				0.06	0.06	1.01	−0.06	0.18
	Grade				−0.08	0.04	−2.24 *	−0.15	−0.01
	Emotion malleability beliefs				0.29	0.03	8.39 **	0.22	0.36
Peer relationship		0.53	0.28	47.06 **					
	Gender				0.03	0.06	0.53 *	0.69	1.40
	Grade				0.03	0.04	0.87	0.18	0.33
	Emotion malleability beliefs				0.25	0.04	6.91 **	0.24	0.41
	Psychological resilience				0.38	0.05	8.38 **	0.27	0.45
Adaptation to school		0.53	0.28	36.44 **					
	Gender				−0.06	0.06	−1.07	−0.18	0.05
	Grade				−0.08	0.03	−2.32 *	−0.15	−0.01
	Emotion malleability beliefs				0.17	0.04	4.44 **	0.09	0.24
	Psychological resilience				0.20	0.05	4.15 **	0.10	0.29
	Peer relationships				0.27	0.04	6.00 **	0.18	0.36

Note. * *p* < 0.05, ** *p* < 0.01.

**Table 5:** The chain mediation effect analysis.

Effect	Path	Effect Value	*SE*	*LL*	*UL*	Relative Mediation Effect (%)
Direct effect	Emotion malleability beliefs → Adaptation to school	0.17	0.04	0.09	0.24	51.84
Indirect effect	Emotion malleability beliefs → Psychological resilience → Adaptation to school	0.06	0.03	0.01	0.14	17.75
	Emotion malleability beliefs → Peer relationships → Adaptation to school	0.07	0.03	0.02	0.14	21.19
	Emotion malleability beliefs → Psychological resilience → Peer relationships → Adaptation to school	0.03	0.01	0.01	0.06	9.22
Total effect	Emotion malleability beliefs → Adaptation to school	0.33	0.04	0.25	0.39	100

## Data Availability

The data presented in this study are available on request from the corresponding author (the data are not publicly available due to privacy).

## References

[B1-behavsci-15-01444] Armstrong A. R., Galligan R. F., Critchley C. R. (2011). Emotional intelligence and psychological resilience to negative life events. Personality and Individual Differences.

[B2-behavsci-15-01444] Asher S. R., Hymel S., Renshaw P. D. (1984). Loneliness in children. Child Development.

[B3-behavsci-15-01444] Bailen N. H., Green L. M., Thompson R. J. (2018). Understanding emotion in adolescents: A review of emotional frequency, intensity, instability, and clarity. Emotion Review.

[B4-behavsci-15-01444] Bolin J. H. (2014). Andrew F. Hayes (2013). Introduction to mediation, moderation, and conditional process analysis: A regression-based approach. New York, NY: The Guilford Press. Journal of Educational Measurement.

[B5-behavsci-15-01444] Burnette J. L., Finkel E. J. (2012). Buffering against weight gain following dieting setbacks: An implicit theory intervention. Journal of Experimental Social Psychology.

[B6-behavsci-15-01444] Chen Y., Xiao S., Li Y., Deng Q., Gao Y., Gao F. (2019). Relationship between shyness and middle school students’ adjustment: A mediated moderation model. Chinese Journal of Clinical Psychology.

[B7-behavsci-15-01444] Congard A., Le Vigouroux S., Antoine P., Andreotti E., Perret P. (2022). Psychometric properties of a French version of the implicit theories of emotion scale. European Review of Applied Psychology.

[B8-behavsci-15-01444] Coyle S., Weinreb K. S., Davila G., Cuellar M. (2021). Relationships matter: The protective role of teacher and peer support in understanding school climate for victimized youth. Child & Youth Care Forum.

[B9-behavsci-15-01444] Dahò M., Monzani D. (2025). The multifaceted nature of inner speech: Phenomenology, neural correlates, and implications for aphasia and psychopathology. Cognitive Neuropsychology.

[B10-behavsci-15-01444] De Castella K., Goldin P., Jazaieri H., Ziv M., Dweck C. S., Gross J. J. (2013). Beliefs about emotion: Links to emotion regulation, well-being, and psychological distress. Basic and Applied Social Psychology.

[B11-behavsci-15-01444] De Castella K., Platow M. J., Tamir M., Gross J. J. (2018). Beliefs about emotion: Implications for avoidance-based emotion regulation and psychological health. Cogntion & Emotion.

[B12-behavsci-15-01444] Eoh Y., Lee E., Park S. H. (2022). The relationship between children’s school adaptation, academic achievement, happiness, and problematic smartphone usage: A multiple informant moderated mediating model. Applied Research Quality Life.

[B13-behavsci-15-01444] Ford B. Q., Gross J. J. (2018). Why beliefs about emotion matter: An emotion-regulation perspective. Current Directions in Psychological Science.

[B14-behavsci-15-01444] Ford B. Q., Lwi S. J., Gentzler A. L., Hankin B., Mauss I. B. (2018). The cost of believing emotions are uncontrollable: Youths’ beliefs about emotion predict emotion regulation and depressive symptoms. Journal of Experimental Psychology.

[B15-behavsci-15-01444] Gangemi A., Dahò M., Mancini F. (2021). Emotional reasoning and psychopathology. Brain Sciences.

[B16-behavsci-15-01444] Glaser M. (1998). Apprehension about communication and human resilience. Psychological Reports.

[B17-behavsci-15-01444] Goodman F. R., Kashdan T. B., Imamoglu A. (2021). Valuing emotional control in social anxiety disorder: A multimethod study of emotion beliefs and emotion regulation. Emotion.

[B18-behavsci-15-01444] Graber R., Turner R., Madill A. (2016). Best friends and better coping: Facilitating psychological resilience through boys’ and girls’ closest friendships. British Journal of Psychology.

[B19-behavsci-15-01444] Haddow S., Taylor E. P., Schwannauer M. (2021). Positive peer relationships, coping and resilience in young people in alternative care: A systematic review. Children and Youth Services Review.

[B20-behavsci-15-01444] Halilova J. G., Ward Struthers C., Guilfoyle J. R., Shoikhedbrod A., van Monsjou E., George M. (2020). Does resilience help sustain relationships in the face of interpersonal transgressions?. Personality and Individual Differences.

[B21-behavsci-15-01444] Hayes A. F. (2013). Introduction to mediation, moderation, and conditional process analysis: A regression-based approach.

[B22-behavsci-15-01444] Hou J. (2016). Development of the scale of school adjustment for junior high school student and its validity and reliability. China Journal of Health Psychology.

[B23-behavsci-15-01444] Hu Y., Gan Y. (2008). Development and psychometric validity of the resilience scale for Chinese adolescents. Acta Psychologica Sinica.

[B24-behavsci-15-01444] Huang S., Cai F., Liu P., Zhang W., Gong W. (2015). Parent-child relationship and school adjustment: The mediating of regulatory emotional self-efficacy. Chinese Journal of Clinical Psychology.

[B25-behavsci-15-01444] Huang Z., Ye B., Yang Q. (2021). Effect of social and emotional competency on social adaptability of middle school students: The chain mediating role of self-esteem and peer relationship. China Journal of Health Psychology.

[B26-behavsci-15-01444] Kashdan T. B., Barrios V., Forsyth J. P., Steger M. F. (2006). Experiential avoidance as a generalized psychological vulnerability: Comparisons with coping and emotion regulation strategies. Behaviour Research and Therapy.

[B27-behavsci-15-01444] Kendziora K., Osher D. (2016). Promoting children’s and adolescents’ social and emotional development: District adaptations of a theory of action. Journal of Clinical Child and Adolescent Psychology.

[B28-behavsci-15-01444] Kim Y., Kim S., Yoon S. (2024). Emotion malleability beliefs matter in emotion regulation: A comprehensive review and meta-analysis. Cognition and Emotion.

[B29-behavsci-15-01444] Klika J. B., Herrenkohl T. I. (2013). A review of developmental research on resilience in maltreated children. Trauma Violence Abuse.

[B30-behavsci-15-01444] Klizienė I., Klizas Š., Čižauskas G., Sipavičienė S. (2018). Effects of a 7-month exercise intervention programme on the psychosocial adjustment and decrease of anxiety among adolescents. European Journal of Contemporary Education.

[B31-behavsci-15-01444] Kneeland E. T., Dovidio J. F. (2020). Emotion malleability beliefs and coping with the college transition. Emotion.

[B32-behavsci-15-01444] Kneeland E. T., Dovidio J. F., Joormann J., Clark M. S. (2016a). Emotion malleability beliefs, emotion regulation, and psychopathology: Integrating affective and clinical science. Clinical Psychology Review.

[B33-behavsci-15-01444] Kneeland E. T., Nolen-Hoeksema S., Dovidio J. F., Gruber J. (2016b). Beliefs about emotion’s malleability influence state emotion regulation. Motivation and Emotion.

[B34-behavsci-15-01444] Llistosella M., Castellví P., García-Ortiz M., López-Hita G., Torné C., Ortiz R., Guallart E., Uña-Solbas E., Carlos Martín-Sánchez J. (2024). Effectiveness of a resilience school-based intervention in adolescents at risk: A cluster-randomized controlled trial. Frontiers in Psychology.

[B35-behavsci-15-01444] Malagoli C., Chiorri C., Traverso L., Usai M. C. (2022). Inhibition and individual differences in behavior and emotional regulation in adolescence. Psychological Research.

[B36-behavsci-15-01444] Mertens E. C. A., Dekovic M., van Londen M., Reitz E. (2022). Parallel changes in positive youth development and self-awareness: The role of emotional self-regulation, self-esteem, and self-reflection. Prevention Science.

[B37-behavsci-15-01444] Romero C., Master A., Paunesku D., Dweck C. S., Gross J. J. (2014). Academic and emotional functioning in middle school: The role of implicit theories. Emotion.

[B38-behavsci-15-01444] Schroder H. S., Kneeland E. T., Silverman A. L., Beard C., Björgvinsson T. (2018). Beliefs about the malleability of anxiety and general emotions and their relation to treatment outcomes in acute psychiatric treatment. Cognitive Therapy and Research.

[B39-behavsci-15-01444] Shen A., Gao W., Gu J., Zhang J., Yu A. (2021). Parent-child communication inconsistency and school adaptation of boarding adolescents: Different mediation of two coping styles and its gender difference. Psychology: Techniques and Applications.

[B40-behavsci-15-01444] Su Y., Yao J., Li L. (2021). The relationship between emotional intelligence and school adaptation of middle school students: The mediating role of psychological resilience. Mental Health Education in Primary and Secondary School.

[B41-behavsci-15-01444] Tamir M., John O. P., Srivastava S., Gross J. J. (2007). Implicit theories of emotion: Affective and social outcomes across a major life transition. Journal of Personality and Social Psychology.

[B42-behavsci-15-01444] Tan Q., Chen Y. (2007). A research for class environments’ influence on junior high school students’ school adjustment. Chinese Journal of Clinical Psychology.

[B43-behavsci-15-01444] Wang B., Lu W., Yuan J., Tian L. (2018). The effects of self-esteem on adolescents’ aggression behavior: The moderating effects of peer relationship. Journal of Shandong Normal University (Natural).

[B44-behavsci-15-01444] Wu Z., Qin Y. (2017). Rural education development in China: An annual report.

[B45-behavsci-15-01444] Xie L., Zou W., Wang H. (2022). School adaptation and adolescent immigrant mental health: Mediation of positive academic emotions and conduct problems. Frontiers in Public Health.

[B46-behavsci-15-01444] Yeager D. S., Dweck C. S. (2012). Mindsets that promote resilience: When students believe that personal characteristics can be developed. Educational Psychologist.

[B47-behavsci-15-01444] Yu X., Wan X., Yitian Z., Duan J., Huang S., Fu Y., Wang J. (2017). Analysis on the correlation between injury of adolescents and school and peer factors. Chinese Journal of Disease Control and Prevention.

[B48-behavsci-15-01444] Yu X., Zhang J. (2005). Resilience: The psychological mechanism for recovery and growth during stress. Advances in Psychological Science.

[B49-behavsci-15-01444] Zhang G., Liang Z., Deng H., Lu Z. (2014). Relations between perceptions of school climate and school adjustment of adolescents: A longitudinal study. Psychological Development and Education.

[B50-behavsci-15-01444] Zhang J., Guo S., Lipp O. V., Wang M. (2023). Emotion malleability beliefs predict daily positive and negative affect in adolescents. Cognition & Emotion.

[B51-behavsci-15-01444] Zhang X., Guo H., Lin D. (2019). A Study on the relationship between parent-child, peer, teacher-student relations and subjective well-being of adolescents. Psychological Development and Education.

[B52-behavsci-15-01444] Zhu S., Ni S., Hamilton K. (2020). Cognition malleability belief, emotion regulation and adolescent well-being: Examining a mediation model among migrant youth. Health Psychology and Behavioral Medicine.

[B53-behavsci-15-01444] Zou H., Qu Z., Ye Y. (2007). The characteristics of teacher-student relationships and its relationship with school adjustment of students. Psychological Development and Education.

